# The Future of Physiology: Cardiac Electrophysiology

**DOI:** 10.3389/fphys.2020.00854

**Published:** 2020-07-15

**Authors:** R. Coronel

**Affiliations:** Department of Experimental Cardiology, Academic Medical Center, Amsterdam, Netherlands

**Keywords:** cardiac electrophysiology, extradisciplinary research, multidisciplinary research, history, future

## Abstract

Is cardiac electrophysiology complete? What are the challenges that are to be met in cardiac electrophysiology and how can we best engage these? These questions will be addressed in view of the progressing subspecialization of the field. A suggested answer lies in multidisciplinary and extradisciplinary approaches.

## Introduction

Burdon Sanderson and Page wrote in 1879: “*…we owe most to the labours of Engelmann, whose researches … on the electromotive properties of the resting heart, and on the electrical changes which immediately follow excitation, leave little more to be done*” (Burdon-Sanderson and Page, 1879). They suggested that almost every question on cardiac electrophysiology had been answered and that the subject had been covered by their predecessor Engelmann (1877). Cardiac electrophysiology was complete. However, almost 120 years later the question whether cardiac electrophysiology was complete was addressed again by de Bakker in his inaugural lecture (de Bakker, 1999). de Bakker (1999) pointed to the many recent and new developments in the field and answered the question in the negative. There were still many challenges ahead in cardiac electrophysiology.

Indeed, after the publications of Burdon Sanderson and Page cardiac electrophysiology has flourished:

– Reentry was described as a potential mechanism underlying clinical arrhythmias (Mines, 1914).
– The Long QT syndromes were recognized as an entity (Jervell and Lange-Nielsen, 1957).
– The role of the autonomic nervous system on cardiac electrophysiology was studied (Schwartz et al., 1977; Shivkumar et al., 2016).
– The transmembrane current underlying pacemaking was discovered (DiFrancesco and Ohba, 1978).
– Ischemia-induced arrhythmias were mechanistically understood (Janse et al., 1980).
– Electrical cardioversion was successfully applied to stop lethal arrhythmias (Lown et al., 1986).
– The intricate relation between anatomy and function of the sinus and atrioventricular nodes was understood (Opthof, 1988; McGuire et al., 1996).
– The histology of myocardial infarction was linked to post-MI VTs (de Bakker et al., 1993).
– The coupling between cardiac mechanics and cardiac electrophysiology has been described (Franz, 1996; Kohl et al., 1998).
– The pulmonary vein myocardium was found as the origin of AF (Haissaguerre et al., 1998).
– The relation between embryological development and arrhythmias was established (Boukens and Christoffels, 2012).

Many more important and life-changing accomplishments of cardiac electrophysiology can be described here. As a spin-off of these insights, pharmacological and device therapies have been designed and new, -molecular biological- therapies are under development. The number of new inventions and breakthroughs have been almost endless and unanswered questions in cardiac electrophysiology remain. A few of these questions are enumerated in the table, some clinically relevant, others with a more basic science origin. The list is not intended to be complete or in order of relevance. Therefore, cardiac electrophysiology appears to have a future. However, is the subspecialization fit to address these questions?

## The Development of Cardiac Electrophysiology as a Subspecialization

Cardiac electrophysiology has a rich history (Lüderitz, 2002) and it is not easy to determine when cardiac electrophysiology constituted a distinct subspecialty of physiology and who was the first cardiac electrophysiologist. Burdon-Sanderson with his work on the cardiac currents resulting from local “injury” probably qualifies as the first cardiac electrophysiologist because he described what we now name a monophasic action potential or MAP (Burdon-Sanderson and Page, 1879; Franz, 1983). His pupil, Waller subsequently recorded the first electrocardiogram in man in 1887 (Waller, 1887). Thus, Burdon Sanderson and Waller shared and transferred their common interest in cardiac electrophysiology and thereby constituted the subspecialty. Nevertheless, the interest of these researchers was much wider than cardiac electrophysiology alone. They also studied the electrophysiology of nerves and plants (the fly trap), the effects of anesthesia and the influence of drugs on muscle contraction, to name but a few topics of their studies (Waller, 1887, 1910).

In the course of time, physiology have undergone further (sub)specialization. Physiology has developed into electrophysiology and cardiac electrophysiology (Weidmann, 1951). This course of (sub)specialization has followed the reductionist approach in science in general. In this approach the researcher uses progressively smaller and simpler models to exclude as many as possible confounding factors and to control all factors excepting the one that he/she is interested in. Indeed, at the same time cardiac electrophysiology evolved from the study of organisms (Waller, 1887) to that of organs (Mines, 1913) multicellular preparations (Kléber and Riegger, 1987) cells, expression systems, subcellular domains (Sanchez-Alonso et al., 2016; Rivaud et al., 2020), genes (Remme et al., 2006), or computer models (Shaw and Rudy, 1997; Hoogendijk et al., 2011). The reductionist development of scientific fields has also transpired in cardiac electrophysiology from which now a branch of cardiac *cellular* electrophysiology exists with dedicated textbooks and a European Working Group of Cardiac Cellular Electrophysiology (EWGCCE) that is part of the European Society of Cardiology. The identification of subcellular microdomains heralds the further subspecialization of the field in cardiac *sub*cellular electrophysiology.

The website of Frontiers in Physiology^1^ is an example of the pedigree of physiology. It mentions 28 subspecialty sections amongst which are Autonomic Neuroscience, Biophysics, Cardiac Electrophysiology, Chronobiology, Clinical and Translational Physiology, Computational Physiology and Medicine, Embryonic and Developmental Physiology, Exercise Physiology, Integrative Physiology, Lipid and Fatty Acid Research, Medical Physics and Imaging, Membrane Physiology, and Membrane Biophysics. These subspecialties are not mutually exclusive. For example, studies on the Brugada Syndrome, a potentially lethal cardiac arrhythmia syndrome occurring at a relatively young age, are related to all of the above enumerated subspecialties of Frontiers in Physiology, but also to Frontiers in Cardiovascular Medicine (subspecialty Cardiac Rhythmology) (Antzelevitch et al., 2005; Hoogendijk et al., 2010; Zhang et al., 2015).

Although the reductionist approach is a powerful tool to identify mechanisms it has potential drawbacks. The results of reductionist studies cannot always be translated to more integrative levels of application. An example in cardiac electrophysiology is the use of the mouse as a model for human electrophysiology. Although the mouse is a good model for many mammalian characteristics including genetics, its ventricular action potential differs from that of man. Genetic studies on ventricular electrophysiology in mice therefore should be translated with caution to human electrophysiology (London, 2001). Another example is that of the use of a small portion of the ventricular wall as a model for the intact heart (Opthof et al., 2007).

The branching process (in progressively more subspecialities) facilitates but may also hinder engagement of the unmet challenges in cardiac electrophysiology. It is clear that within the domains of knowledge and techniques pertaining to cardiac electrophysiology we are more likely capable of finding an answer to the question “How does the Purkinje network relate to arrhythmias?” than to the question “How do we build a (human) heart?” (Table 1). Thus, it appears that physiology has branched into too many sub- and infra-specializations to allow a more generalist ‘integrative’ approach. How can we best address the unanswered questions?

**TABLE 1:** Some questions!

– How do we build a (human) heart? – How can we prevent sudden death? – How do we assess the risk of arrhythmias in patients? – How do we diagnose and manage congenital arrhythmia syndromes? – What is the relation between aging or prenatal development and arrhythmogenesis? – Can we (non-invasively) map activation and repolarization of all myocardial components of the heart? – What is the difference between the heart from a man and that of a woman? – How can we best optimize the contraction sequence in diseased hearts? – Do ion-transporting proteins have other (gene regulating, trafficking) functions? – How does the Purkinje network relate to arrhythmias? – What is the effect of obesity and other chronic diseases on arrhythmogenesis? – What is the mechanism of J-wave syndromes? – How do we prevent atrial fibrillation? – Can we increase the total number of heart beats in a life?

## Integrative Research

In view of the immense growth of published knowledge in the course of the 20th century (Coronel, 2020) it would appear to be impossible to combine this knowledge in one person and to become a generalist again. Therefore, we need to resort to collaboration with other scientists to complement the missing knowledge. Multidisciplinary research, the collaborative effort of scientists of various specializations and disciplines to solve a common problem, has been very successful.

Purkynje was the founder of the modern physiological laboratory and already was aware of the power of combining multiple disciplines (Travnickova, 1987). Multidisciplinarity was also applied by Dirk Durrer who combined the disciplines medicine, biology, biochemistry, electronics and physics (and a well-equipped workshop) for his research on cardiac electrophysiology and arrhythmias (Durrer et al., 1970). Many electrophysiological labs have followed this example and now also incorporate molecular biology, genetics, imaging, artificial intelligence, and developmental biology (stem cell biology) as disciplines to support electrophysiological research. The added value of the collaborations stems from the novel insights and techniques obtained in each discipline or from the discovery of new (genetic) syndromes (Early repolarization syndrome and Brugada syndrome). These insights/techniques encompass -amongst others- new model systems (for example, pluripotent stem cells), enlarged computing power, and new imaging modalities (scanning ion conductance microscopy, near infrared optical mapping in explanted human hearts) (Hansen et al., 2018). It remains a challenge to select the best possible fitting (animal) model to address a research also in more integrative modes of research. The best model for human (electrophysiology) remains the human, although this often is not ethically appropriate.

Multidisciplinary research potentially has an impact that reaches far outside the field of cardiac electrophysiology. In most examples of multidisciplinary research, the central research question or hypothesis is generated from a co-ordinating specialization to which the contributors from the other disciplines apply their knowledge. The question ‘How do we diagnose and manage congenital arrhythmia syndromes?’ can be solved by asking geneticists and molecular biologists to join a team of cardiologists.

Extradisciplinary research constitutes a particular mode of multidisciplinary research. This describes the scientific study of a research question or hypothesis generated in a different discipline (or subspecialty). Extradisciplinary research is a way to overcome the drawbacks of a too far progressed specialization and a way to a more integrative research approach of physiology. When cardiac electrophysiology was in its early days, the various disciplines were combined in a single person and extradisciplinary research was performed as a matter of course. A problem in plant physiology could thus be solved by applying knowledge from the mammalian heart and vice versa. Extradisciplinary input to cardiac electrophysiology can be anticipated from mathematics (inverse electrocardiography), developmental biology (congenital arrhythmia syndromes), and neurology (arrhythmia mechanisms). On the other hand, cardiac electrophysiology may impact on heart failure management (pacing), and cardiac repair by biological tissue constructs.

Cardiac arrhythmias are often associated with cardiac and extracardiac morbidities like obesity, hypertension, diabetes, atherosclerosis and neurological disorders. The exact contribution of these co-morbidities to cardiac arrhythmogenesis is often not known. A solution to these question lies in unraveling the interaction between extracardiac factors and cardiac electrophysiology by asking endocrinologists, immunologists and neurologists to address cardiac arrhythmogenesis and by stimulating cardiac electrophysiologists to perform extracardiac studies in extradisciplinary approaches.

Thus, if we wish to advance cardiac electrophysiology into the future, we need to allow a ‘generalist’ view on the subject by invoking other disciplines to operate on our turf. On the other hand, cardiac electrophysiology may well be fit to address questions on other fields of research.

## Conclusion

Although cardiac electrophysiology has a rich history it is far from complete. There are still many questions that need to be resolved. However, cardiac electrophysiology has seen the emergence of subspecialties that may hinder a more generalist tackling of research. A plea is made for a multidisciplinary research and for extradisciplinary research in particular. As with all collaborative projects, the success of extradisciplinary research depends on mutual trust and clear communication between the scientists of the various disciplines.

## Author Contributions

The author confirms being the sole contributor of this work and has approved it for publication.

## Conflict of Interest

The author declares that the research was conducted in the absence of any commercial or financial relationships that could be construed as a potential conflict of interest.

## Footnotes

1 www.frontiersin.org
